# VIVA1: a more invasive subclone of MDA-MB-134VI invasive lobular carcinoma cells with increased metastatic potential in xenograft models

**DOI:** 10.1038/s41416-022-01778-7

**Published:** 2022-03-22

**Authors:** Victoria Allen, Josée Coulombe, Huijun Zhao, Lauren M. Kreps, David P. Cook, Benjamin Pryce, Mark Clemons, Barbara C. Vanderhyden, Douglas A. Gray, Christina L. Addison

**Affiliations:** 1 Program for Cancer Therapeutics, Ottawa Hospital Research Institute, Ottawa, K1H 8L6 ON Canada; 2 Department of Biochemistry, Microbiology and Immunology, University of Ottawa, Ottawa, K1H 8M5 ON Canada; 3 Department of Cellular and Molecular Medicine, University of Ottawa, Ottawa, K1H 8M5 ON Canada; 4 Department of Medicine, University of Ottawa, Ottawa, K1H 8M5 ON Canada

**Keywords:** Breast cancer, Cancer models, Metastasis

## Abstract

**Background:**

Invasive lobular carcinoma (ILC) is the second most common type of breast cancer. As few tools exist to study ILC metastasis, we isolated ILC cells with increased invasive properties to establish a spontaneously metastasising xenograft model.

**Methods:**

MDA-MB-134VI ILC cells were placed in transwells for 7 days. Migrated cells were isolated and expanded to create the VIVA1 cell line. VIVA1 cells were compared to parental MDA-MB-134VI cells in vitro for ILC marker expression and relative proliferative and invasive ability. An intraductally injected orthotopic xenograft model was used to assess primary and metastatic tumour growth in vivo.

**Results:**

Similar to MDA-MB-134VI, VIVA1 cells retained expression of oestrogen receptor (ER) and lacked expression of E-cadherin, however showed increased invasion in vitro. Following intraductal injection, VIVA1 and MDA-MB-134VI cells had similar primary tumour growth and survival kinetics. However, macrometastases were apparent in 7/10 VIVA1-injected animals. Cells from a primary orthotopic tumour (VIVA-LIG43) were isolated and showed similar proliferative rates but were also more invasive than parental cells. Upon re-injection intraductally, VIVA-LIG43 cells had more rapid tumour growth with similar metastatic incidence and location.

**Conclusions:**

We generated a new orthotopic spontaneously metastasising xenograft model for ER+ ILC amenable for the study of ILC metastasis.

## Background

Invasive lobular carcinoma (ILC) is the 2nd most common type of breast cancer after invasive ductal carcinoma (IDC) and accounts for 10–15% of all diagnosed breast cancer, with metastatic disease occurring in 20–30% of patients [1–4]. ILC is classified as luminal A or B [1–5], but its growth pattern makes it difficult to palpate lesions [6] which are not readily seen on screening mammography thereby leading to delays in diagnosis [7]. Patients with ILC thus frequently present at more advanced stage disease than those with IDC [8, 9]. Although generally considered to be low-risk breast cancer, ILC responds poorly to chemotherapy [10] with fewer pathological complete responses to preoperative chemotherapy [11] and higher recurrence rates after surgery [12] compared to IDC. Like IDC, ILC most often spreads to bone [13, 14], but also spreads to unique sites including the spleen, ovary, gastrointestinal tract, peritoneum and skin [15, 16].

ILC is characterised by the specific loss of E-cadherin expression or function [17, 18], however, it has been shown that this is insufficient to drive ILC tumour development in preclinical models [19]. Although ILC was recently shown to be genetically and histologically distinct from IDC [20–22], ILC patients are clinically treated in the same manner as IDC patients. This is despite the fact that ILC patients are more likely to have higher nodal involvement, multiple concurrent metastatic sites and worse overall survival than IDC patients [8, 22]. Given the worse prognostic outcome for ILC patients and the lack of response to chemotherapy [8, 23], it is crucial to identify factors driving ILC metastasis so that novel, effective ILC therapies may be developed.

The study of factors driving metastatic ILC has been confounded by a lack of available preclinical tools with which to study this process. It has been reported that many of the available ILC cell lines are not invasive in vitro [24], and to date only one cell line, IPH926, has been shown to grow in xenograft models [25]; however, it is a triple-negative tumour cell line and thus may not be representative of the majority of ILC patient tumours which are oestrogen receptor-positive (ER+). Of the described ILC transgenic models [19, 26–28], two have confirmed the development of ILC micrometastases in ~30–70% of animals [19, 27]. Tumour cells isolated from one of these transgenic models (*Wcre;Cdh1;*^*F/F*^*Trp53*^*F/F*^) developed metastatic spread upon orthotopic transplantation of isolated tumour cells, however, it was reported that most tumour cells were ER− [28]. Recently, despite poor rates of successful engraftment of ER+ breast cancer patient tumours in PDX models [29], four patient-derived xenograft (PDX) models for ILC have been reported to develop metastasis, with two of these being ER−. Thus, currently available metastatic ER+ ILC research tools remain limited.

In order to better identify pathways responsible for promoting the metastasis of ILC and to effectively test therapeutic strategies designed to block or treat ILC metastasis, we sought to develop a xenograft model with the ability to spontaneously metastasize from the orthotopic mammary gland site. To this end, we now describe the isolation and characterisation of an invasive subclone from the parental lobular cell line MDA-MB-134VI. The MDA-MB-134VI cell line was originally isolated from pleural effusion samples from a 47-year-old Caucasian female [30] and has subsequently been shown to be ER+, progesterone receptor (PR)−, HER2 not amplified and E-cadherin− [31–33]. Although it was previously suggested that this cell line was non-invasive in vitro [24], we have isolated a more invasive subclone of this cell line which we have named the VIVA1 cell line. When tested in an orthotopic xenograft model, VIVA1 cells, along with the parental MDA-MB-134VI cells readily grew in mammary ducts following intraductal injection as previously described [34], however, VIVA1 cells spontaneously metastasised to distal sites. Herein, we describe the isolation and characterisation of VIVA1 cells which represent a new orthotopic spontaneously metastasising ER+ ILC preclinical in vivo model.

## Materials and methods

### Cell lines and reagents

MDA-MB-134VI, MDA-MB-330 and UACC-3133 cells were purchased from ATCC (American Type Culture Collection, Manassas, VA). MDA-MB-134VI cells were adapted from air culture to 5% CO_2_ at 37 °C. MDA-MB-134VI cells were grown in 1:1 ratio of DMEM and Leibovitz’s L-15 medium supplemented with 2 mM l-glutamine (Gibco, ThermoFisher Scientific, Ottawa, ON) and 20% foetal bovine serum (FBS, Hyclone, ThermoFisher Scientific, Ottawa, ON). MDA-MB-330 cells were grown in 1:1 ratio of DMEM and Leibovitz’s L-15 medium supplemented with 2 mM l-glutamine (Gibco, ThermoFisher Scientific, Ottawa, ON), 20% foetal bovine serum (FBS, Hyclone, ThermoFisher Scientific, Ottawa, ON), 30 ng/mL recombinant human epidermal growth factor (EGF) (Invitrogen, Gibco, ThermoFisher Scientific, Ottawa, ON), 0.016 mg/mL insulin solution from bovine pancreas (Sigma-Aldrich, Oakville, ON) and 2 mM l-glutathione (Sigma-Aldrich, Oakville, ON). The UACC-3133 cell line was grown in Leibovitz’s L-15 with 5% FBS, 5 μg/mL catalase from bovine liver (Sigma-Aldrich, Oakville, ON), 0.01 mg/mL human transferrin (Sigma-Aldrich, Oakville, ON), 0.01 mg/mL insulin solution from bovine pancreas, 3.6 μg/mL hydrocortisone (Sigma-Aldrich, Oakville, ON) and 2 mM GlutaMAX™ (Gibco, ThermoFisher Scientific, Ottawa, ON). The IPH926 cell line [35] was a kind gift of Dr. Christgen and was grown in Roswell Park Memorial Institute medium containing 2.05 mM l-glutamine (RPMI-1640) (GE Healthcare LifeSciences, Mississauga, ON) supplemented with 10 μg/mL insulin solution from bovine pancreas, 2.5 g/L D-(+)-glucose (Sigma-Aldrich, Oakville, ON), 10 mM N-2-hydroxyethylpiperazine-N-2ethane sulfonic acid (HEPES) (Gibco, ThermoFisher Scientific, Ottawa, ON) and 1 mM sodium pyruvate (Gibco, ThermoFisher Scientific, Ottawa, ON). Cells were tested for mycoplasma monthly to confirm mycoplasma negative cells were used for all experiments. For western blot analysis, primary antibodies used were as follows: mouse monoclonal anti-human E-cadherin antibody (610181, BD Pharmingen, BD Biosciences, Mississauga, ON); mouse monoclonal anti-human ERα (6F11) antibody (MA1-80216, Invitrogen, ThermoFisher Scientific, Ottawa, ON); rabbit monoclonal anti-human HER2/ErB2 (29D8) antibody (2165, Cell Signalling Technology Inc., New England Biolabs Ltd., Whitby, ON); goat anti-firefly luciferase antibody (AB181640, Abcam, Toronto, ON); mouse monoclonal anti-human β-actin antibody (A5316, Sigma-Aldrich, Oakville, ON). Secondary antibodies used included: peroxidase AffiniPure goat anti-mouse IgG (H+L) (115-035-146, Jackson ImmunoResearch Inc., West Grove, PA) and peroxidase AffiniPure goat anti-rabbit IgG (H+L) (111-035-144, Jackson ImmunoResearch Inc., West Grove, PA) and biotinylated rabbit anti-Goat (H+L) IgG (BA-5000, Vector Laboratories, Burlingame, CA).

### Invasion and migration assays

Matrigel-coated 8.0-μm membrane pore size transwell plates (Corning, Corning, NY) were used to test cell invasion, which was performed using an adapted recommended manufacturer’s protocol. Prior to plating cells, the invasion chambers were rehydrated for 2 h in culture media containing 1% FBS. A total of 5 × 10^4^ cells were then plated in the top chamber transwell insert in culture media containing 1–5% FBS. Culture media containing 20% FBS was placed in the bottom chamber to create a gradient of FBS, and cells were allowed to invade for either 48 h or 7 days. Media from inside the inserts was then aspirated, and a cotton swab was used to remove non-invading cells remaining on the surface of the upper side of the membrane. Transwell inserts were then placed in ~500 μL of methanol for 10–30s to fix cells on the membrane which was then transferred to a well containing 0.5% Crystal Violet solution (500 mg Crystal Violet (ThermoFisher Scientific, Ottawa ON), 25 mL 100% methanol, 75 mL ddH_2_O) for 30–60s. Membranes were then washed three times with water to remove excess stain. For enumeration of invaded cells in Matrigel-coated transwell invasion assays, five random fields of view were imaged for each membrane at ×10 using a Nikon Eclipse TE2000-U (Nikon, Mississauga, ON) and the number of invading cells was counted using the ImageJ (National Institutes of Health, Bethesda, MA) Cell Counter plugin (Kurt De Vos, University of Sheffield, Sheffield, UK). Alternatively, membranes were dried overnight after staining was completed, removed from the Transwells and mounted on slides under coverslips using Vecta Mount (Maravai LifeSciences, San Diego, CA). The slides were then imaged using the Aperio ScanScope system (Leica Biosystems, Concord, ON), and the number of cells was counted from five representative images at the ×20 field of view using the Aperio ImageScope software (Leica Biosystems, Concord, ON). After counting the number of migrating/invading cells, the data was either normalised to the control treatments and plotted, or raw counts subsequently plotted.

### Isolation of cells from invasion assay chambers

MDA-MB-134VI cells were placed in invasion chambers as described above and following incubation for 7 days, cells that had invaded to the bottom of the membranes were isolated and pooled from a total of 12 chambers. Briefly, to collect invaded cells, the media was aspirated from the invasion chambers, and the non-invading cells on the top side of the membrane were scrubbed off using sterile cotton swabs. The invasion chambers were then transferred into a 24-well plate containing 1 mL of 1× DPBS per well, to wash the membranes, which were then placed into wells containing 0.05% trypsin to remove cells that had invaded through to the bottom side of the membrane. After trypsinization, the invasion chamber was removed and culture media containing 20% FBS was added to neutralise the trypsin. The media from all wells was then collected and combined followed by centrifugation to pellet cells. Cells were resuspended in growth media and plated in two wells of a 24-well plate which were then expanded to generate the pooled VIVA1 subclone.

### Cell growth assay

To assess cell growth and viability, cells were harvested by trypsinization and 2.5 × 10^4^ cells/well were seeded in duplicate in a 24-well plate. To assess growth kinetics, cells in duplicate wells were independently harvested by trypsinization every 2 days over time and viable cells were counted using Trypan blue solution (Sigma-Aldrich, Oakville, ON) staining and enumeration with the Vi-Cell XR (Beckman Coulter, Indianapolis, IN). Duplicate values were then averaged and graphed. Alternatively, cell viability was assessed using alamarBlue (ThermoFisher, Ottawa, ON) according to the manufacturer’s instructions. Briefly, 5000 cells/well were seeded in each of 8 wells of a 96-well microtiter plate and allowed to adhere overnight. The next day (time 0), the number of viable seeded cells was assessed following the addition of alamarBlue and absorbance measured at Ex 530 nm Em 590 nm. Cell viability was measured every 2 days for a period of 8 days and data normalised to time 0 for graphical representation.

### Western blot

To generate total protein lysates, cells were washed twice at room temperature with 1× Dulbecco’s phosphate-buffered saline (DPBS) followed by lysis in radioimmunoprecipitation assay (RIPA) buffer containing Protease Inhibitor Cocktail (2 mM 4-(2-aminoethyl)benzenesulfonyl fluoride hydrochloride (AEBSF), 0.3 μM Aprotinin, 116 μM Bestatin, 14 μM E-64, 1 μM Leupeptin, 1 mM EDTA; Sigma-Aldrich, Oakville, ON). Lysates were transferred to an Eppendorf tube, vortexed and incubated on ice for 30 min, followed by centrifugation for 15 min at 13500 RPM at 4 °C and supernatants stored at −80 °C. Total protein in lysates was quantified using the Bio-Rad Protein Assay Dye Reagent Concentrate (Bio-Rad, Mississauga, ON), and absorbance measured at 595 nm using the BioMate 3 (ThermoFisher Scientific, Ottawa ON) which then provided a protein quantification in mg/mL after performing a curve linear regression 2nd order and interpolation of a standard curve generated from known quantities of BSA. For subsequent analysis by western blot, 50 μg of total protein was mixed with 1× ß-mercaptoethanol based sample loading buffer followed by incubation at 100 °C for 5 min to denature proteins. Samples were then subjected to electrophoresis in 10% Tris-polyacrylamide gels in glycine based running buffer at 140 V in the Bio-Rad western apparatus system. A BLUeye Prestained Protein Ladder (FroggaBio Inc, Toronto, ON) was included in a separate lane of the gel, to facilitate the identification of protein molecular weights. Proteins were then transferred to Immobilon-P PVDF membrane (Millipore Ltd., Etobicoke, ON) by electrophoresis, and non-specific sites on the membrane were blocked in 5% blocking solution consisting of either skim milk or bovine serum albumin (BSA) in 1× TBST (TBS with 1% Tween-20) for ~1 h at 4 °C on a platform rocker. Membranes were then incubated with a specific primary antibody diluted in either 5% skim milk or 5% BSA in 1× TBST and incubated overnight at 4 °C on a platform rocker, followed by washing three times with 1× TBST for approximately 5 min each. Membranes were then incubated with appropriately diluted secondary antibody for 1 h at room temperature followed by five washes with 1× TBST. Protein antibody conjugates were then visualised following the addition of Bio-Rad Clarity Western ECL Substrate (Bio-Rad, Mississauga, ON) and subsequently imaged using either the GeneGnome Syngene Bio Imaging imager (Syngene, Frederick, MD) or HyBlot CL Autoradiography Film (Denville Inc., Saint-Laurent, QC) and JP33 JPI Automatic X-ray film processor (JPI Healthcare, Plainview, NY).

### RNA-seq

RNA was isolated from VIVA1 or MDA-MB-134VI cells using the RNeasy Kit (Qiagen, Toronto ON) and RNA concentration in ng/μl determined by measuring absorbance using a ND-1000 Nanodrop Spectrophotometer (ThermoFisher Scientific, Waltham, MA). RNA-seq was performed at the Biomedical Research Core at the University of British Columbia (Vancouver, BC). Briefly, RNA quality was confirmed using the Agilent 2100 Bioanalyzer, and then used to generate cDNA libraries following the standard manufacturer’s protocol for the NEBnext Ultra ii Stranded mRNA (New England Biolabs, Whitby, ON). Sequencing was performed on the Illumina NextSeq 500 (Illumina, San Diego, CA) with paired-end 42 bp × 42 bp reads. Transcript quantification for each sample was then performed using Kallisto (v0.45.0) [36] with the GRCh38 build of the human transcriptome and the -b 50 bootstrap option. The R package Sleuth (v0.30.0) [37] was then used to model gene expression changes between cell lines and the Wald’s test was used to test for differential expression. Gene sets (GO Terms) enriched in the differentially expressed genes (adjusted *P* < 0.05, |model beta coefficient| >0.5) were assessed using DAVID (https://david.ncifcrf.gov). RNA-seq data can be accessed at https://www.ncbi.nlm.nih.gov/geo/query/acc.cgi?acc=GSE197155.

### RNA extraction and qRT-PCR

RNA was extracted from cells using the RNeasy kit (Qiagen, Montreal, QC), quantified following measurement of absorbance at 260 nm on a spectrophotometer and stored at −80 °C. Complementary DNA (cDNA) was synthesised by combining 1 µg of total RNA with 10mM dNTP, and Oligo (dT) (Invitrogen, ThermoFisher, Ottawa ON) to prime the reaction. The sample was then heated at 65 °C for 5 min. A total of 4 µL of 5× First-Strand Buffer, 1 µL of RNAse out, and 2 µL of 0.1 M dithiothreitol (DTT) were then added to each tube, and the samples were heated at 37 °C for 2 min. Subsequently, 1 μL of Moloney murine leukaemia virus (M-MLV) reverse transcriptase (Invitrogen, ThermoFisher, Ottawa, ON) was then added to each sample. The samples were then incubated at 37 °C for 50 min, followed by inactivation at 70 °C for 15 min. cDNA was stored at −20 °C until further use.

Polymerase chain reaction (PCR) was performed using cDNA, individual primers (forward and reverse) for specific genes of interest, nuclease-free water and RT SYBR Green ROX qPCR Mastermix (Qiagen, Montreal, QC), using the 7500 Fast Real-Time PCR System (Applied Biosystems by Life Technologies, Carlsbad, CA). The reaction was completed with the following steps, denaturation at 95 °C for 10 min, followed by PCR for 40 cycles, using denaturation of 95 °C for 15 s and elongation at 60 °C for 1 min. The following primers were used: CAPG (forward: CAAACTCTGGAAGACCTTGGCT, reverse: CACTAGGTAGGAGTCCCCCG), SNCG (forward: TCAGTGGCCGAGAAGACCAA, reverse: GGCCTGTAGCCCTCTAGTCT), HMGCS2 (forward: TACCACCAATGCCTGCTACG, reverse: TGGGACGAGCATTACCACTG), ROR2 (forward: TCCTCGAAGTGGACCCGT, reverse: AAGGGGTCCTAAAGGGTCGT), CPXL2 (forward: AGAAGGAACCACCTCGTGGA, reverse: GCTCTGGTTAGCGTGCCTTA), KCND3(forward: GGCTGCTGGGCGCTT, reverse: TGCGGTAGAAGTTGAGCACG), and β-actin (forward: CCAACCGCGAGAAGATGA; reverse: CCAGAGGCGTACAGGGATAG) which was used for normalisation.

### Luciferase expression

MDA-MB-134VI and VIVA1 cells were transfected to express luciferase. pLentiPGKBlastV5-Luc (19166, Addgene, Watertown, MA) was cotransfected with pCMV-dr8.2-dvpr and pCMV-VSV-G (Addgene, #8455 and 8454, respectively) in 293T cells and isolation of viral vectors from cell supernatants was performed. Lentivirus was then used to infect ILC cells and luciferase expression confirmed using the dual-luciferase reporter assay system (Promega, Madison, WI) with detection of luminescence using a Fluoroskan plate reader (Thermofisher, Ottawa, ON).

### Intraductal tumour xenografts

To test in vivo tumour growth of cell lines, CD-1 Nude (086, Charles River Laboratories, Wilmington, MA) or NSG mice (005557, Jackson Labs, Sacramento, CA) were injected intraductally with 1 × 10^5^ cells per gland in the inguinal or abdominal mammary glands using approaches similar to those previously described [34]. Animals were randomly assigned to cages upon receipt and cages randomly assigned to receive an injection with the various tumour cell lines. As a new model was being tested a priori sample size calculation was not possible, thus feasibility and outcome determined sample size. Investigators were not blinded to the experimental condition of each animal cage. For injections, animals were anaesthetised with a ketamine/xylaxine mixture, and placed in a supine position. Tumour cells were injected in a total of 15 μl (10 μl in the lowest inguinal glands) using a Hamilton syringe into four abdominal and four inguinal mammary glands via insertion into the nipple duct while being visualised under a dissecting microscope. In some cases, animals were only injected in 4–6 of these glands due to technical issues or animals awaking from anaesthesia prior to completing injection of all eight glands. Injected animals were monitored over time visually and using tumour imaging on the IVIS Spectrum (Perkin Elmer, Guelph, ON) as described [38, 39]. All animal experiments were performed and approved by the University of Ottawa Animal Care Committee and conformed to the guidelines set by the Animals for Research Act and the Canadian Council on Animal Care’s Guide to the Care and Use of Experimental Animals (Vol. 1, 2nd ed., 1993), and meet standards of practice in the disciplines of laboratory animal science and laboratory animal veterinary medicine. Under these guidelines, clinical endpoints adhered to and used to determine survival endpoint included: Signs of pain or distress (including withdrawal, biting response, piloerection, hunched back, sunken eyes and abdomen, dehydration, weight loss, lethargy), signs of post renal failure or respiratory distress, ambulatory difficulties, tumour ulceration or tumour growth >2 cm in one dimension.

### Isolation of cell lines from tumours

At the clinical endpoint, animals were euthanized and tumours dissected. Portions of tumours were minced and treated with 2.5 μg/ml collagenase in DPBS with shaking for 1 h at 37 °C. Cells were removed, and fresh collagenase solution added for incubation for an additional 1hr at 37 °C. Isolated single cells were then plated in tissue cultures dishes in 1:1 DMEM:Leibovitz L-15 with 20% FBS and expanded for use in downstream applications.

### Immunohistochemistry

At the clinical endpoint, portions of resected tumours were also fixed in 10% neutral buffered formalin and paraffin-embedded. Embedded tissue was sectioned at the 5-μm thickness and mounted on slides which were then stained with hematoxylin and counterstained with eosin as described [40]. Slides were then scanned on an AxioScan slide scanner (Zeiss, North York, ON), and images were processed and analysed using the Zen Blue software package (Zeiss, North York, ON).

### Data presentation and statistics

Data were graphed by choosing relevant types of graphs through GraphPad Prism 5 and 6 software (GraphPad Software, San Diego, CA). The appropriate statistical analysis was selected and calculated using the included GraphPad Prism software (GraphPad Software, San Diego, CA). One-way ANOVA with Tukey’s multiple comparisons was used to analyse datasets with multiple groups (GraphPad Statistics Guide, GraphPad Software, 2017). Comparison of two groups, control vs. treatment, was analysed using an unpaired *t* test with Welch’s correction (GraphPad Statistics Guide, GraphPad Software, 2017). A two-way ANOVA with Bonferroni multiple comparisons was used to analyse multiple groups and factors (GraphPad Statistics Guide, GraphPad Software, 2017). Variance was assessed using F-testing. All graphs are plotted as mean values unless otherwise specified (GraphPad Statistics Guide, GraphPad Software, 2017). All error bars represent the standard error of the mean (SEM), and levels of significance is indicated with *, with the corresponding *P* value found in the figure’s description.

## Results

### Characterisation of ILC cell line invasion

**Fig. 1:**
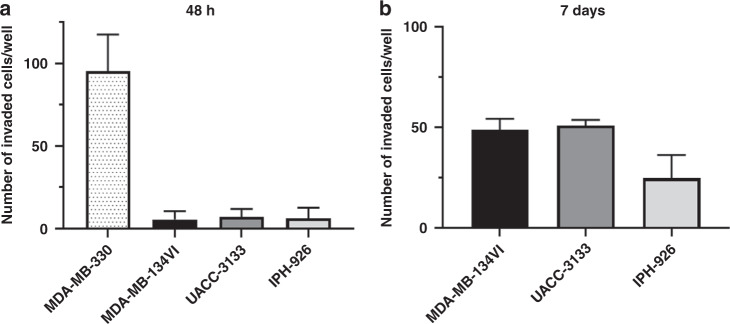
ILC cell lines are generally minimally invasive. The ILC cell lines, MDA-MB-330, MDA-MB-134VI, UACC-3133 and IPH926 were plated on the surface of Matrigel-coated transwell invasion chambers in their respective growth media with reduced FBS (5%) and were allowed to migrate towards increased FBS concentrations (20%) for (**a**) 48h or (**b**) 7 days. Invading cells were stained with crystal violet, counted from five representative images and plotted with the SEM. We considered MDA-MB-330 as an invasive ILC cell line as they were able to invade within 48 h of plating in an invasion chamber, while all other lines tested were considered minimally invasive (*n* = 3 biological replicates, each with duplicate technical replicates).

Based on previous studies suggesting that ILC cell lines were not invasive in Matrigel-coated transwell models in vitro [24], we initially tested the invasive ability of four available ILC cell lines, MDA-MB-330, MDA-MB-134VI, IPH926 and UACC-3133 to confirm these findings. Cells were seeded into upper chambers of Matrigel-coated transwells and directed to migrate towards lower chambers containing media with higher serum concentration as described in the Methods section. Transwell filters were removed, stained using crystal violet and enumerated for migrated cells after 48 h or 7 days. We found that only MDA-MB-330 cells were able to effectively invade through transwell membranes after 48 h of incubation (~80–100 cells on average), while all other cell lines had minimal to no cell invasion (Fig. 1a). Minimally invasive cells did however show increased cell numbers invading through transwells after 7 days of incubation (Fig. 1b), however, quantity remained generally low (ie 20–50 cells per membrane).

### Isolation and characterisation of invasive subclones from MDA-MB-134VI cells

**Fig. 2:**
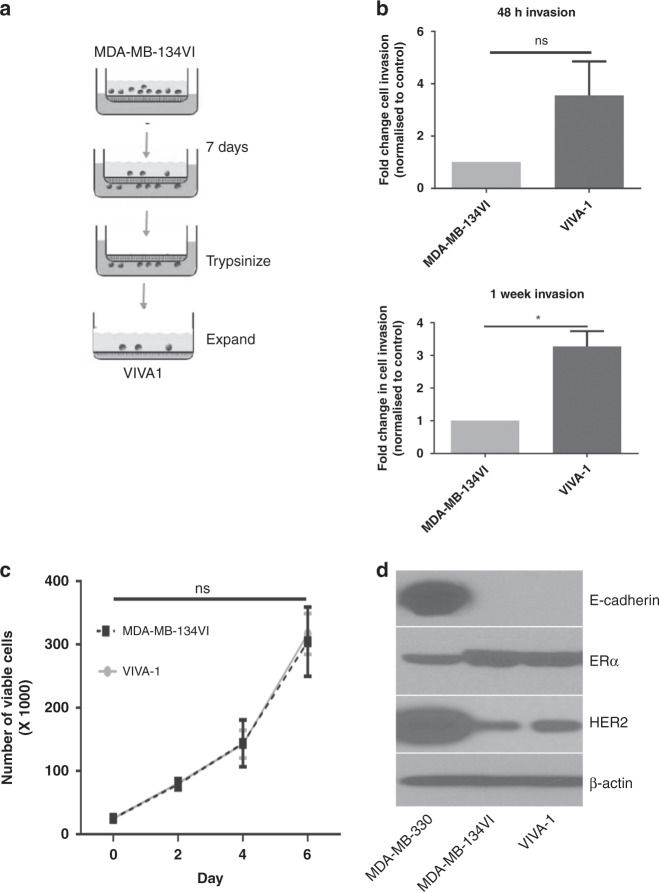
Isolation and characterisation of a more invasive subclone from MDA-MB-134VI. **a** MDA-MB-134VI cells were seeded into Matrigel-coated transwell invasion chambers and allowed to migrate for 7 days, at which time migrated cells were isolated from membranes by trypsinization and expanded to generate the VIVA1 subclone. **b** Invasion of VIVA1 subclone was compared to parental MDA-MB-134VI cells using Matrigel-coated transwell invasion chambers and incubated for 48 h (top panels) or 7 days (bottom panels), at which time cells were counted in five fields of view in duplicate chambers. Graphs represent mean and SEM of migrated cells for *n* = 3 biological replicates. * represents *P* value ≤0.05 using unpaired *t* test with Welch’s correction. **c** Cell growth and viability over time were measured for VIVA1 cells compared to control parental MDA-MB-134VI cells using trypan blue exclusion and counting of cells on the ViaXR cell counter. Graphs represent the mean number of viable cells per well with associated SEM in duplicate wells for *n* = 3 biological replicates. Significance was tested using a two-way ANOVA with Sidak’s multiple comparison testing (ns represents *P* value >0.05). **d** Protein lysates from plated cells were generated and 50µg of protein was subjected to western blot analysis for the proteins indicated. Western blot detection of β-actin was used as the loading control.

Although MDA-MB-330 was more invasive compared to other cell lines, we felt it insufficient to study a single-cell line to gain insight into pathways driving ILC metastasis and thus attempted to isolate invasive cell subclones from the minimally invasive ILC cell line MDA-MB-134VI to increase our sample size of invasive cell lines with which to study this process. We chose MDA-MB-134VI as it was more representative of the ER+ classical ILC subtype that affects 80% of ILC patients and is often used as a model to study ILC [41–45]. Moreover, during the course of our studies, it was suggested that this cell line could grow in mammary ducts following intraductal injection [34], suggesting it may have some utility as a xenograft model for ILC. We thus set up Matrigel-coated transwells as described in the Methods section with MDA-MB-134VI cells and allowed cells to migrate for 7 days, at which time cells were isolated from the bottom of transwell membranes, pooled and expanded (Fig. 2a). We termed the resulting isolated subclone VIVA1. We next tested the relative invasive ability of VIVA1 cells compared to the parental MDA-MB-134VI cells at both 48 h and 7 days post seeding. The VIVA1 subclone had increased invasion at both time points examined when compared to the basal invasion rates of MDA-MB-134VI reaching statistical significance in the 7-day invasion assay (Fig. 2b). We also assessed the relative growth rates of cells by counting viable cell numbers over time using trypan blue exclusion. The growth rate of VIVA1 was almost identical to that of the parental MDA-MB-134VI cell line (Fig. 2c). We also assessed the levels of expression of relevant ILC markers by western blot. VIVA1 cells were found to have similar levels of expression of ERα and HER2 as parental MDA-MB-134VI and maintained the absence of E-cadherin expression as expected (Fig. 2d).

**Fig. 3:**
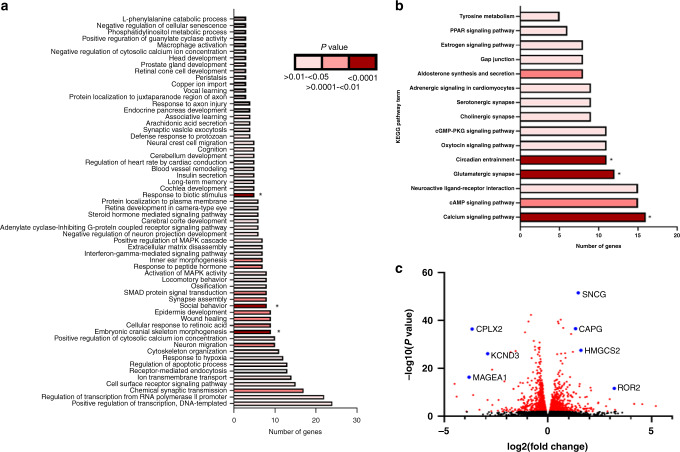
RNA-seq analysis shows the altered gene expression in VIVA1 cells associated with increased cell growth and invasion. RNA was isolated from cell lines using the mRNeasy kit and subjected to next-generation RNA-seq using the Illumina NextSeq as described in 'Materials and methods'. Following sequence analysis and determination of differential gene expression, targets which showed statistically significant log fold changes >0.5 or <−0.5 were submitted to the DAVID online pathway analysis to generate the GoTerm list illustrated in (**a**) with *P* values colour-coded as indicated and those with FDR <0.05 indicated with *. **b** Significantly altered gene lists were also subjected to KEGG pathway analysis and graphically represented with *P* values colour-coded as indicated and those with FDR <0.05 indicated with *. **c** Log2 differential expression was plotted against the associated inverse log 10 *P* value to generate volcano plots. Red dots represent those gene targets with significantly different expression (i.e. *P* value <0.05) between VIVA1 and MDA-MB-134VI. Blue dots are also significantly differentially expressed genes but are highlighted for attention.

We also performed RNA sequencing (RNA-seq) analysis on two independently isolated RNA extracts to compare gene expression in the VIVA1 subclone compared to its parental MDA-MB-134VI cell line (see Supplementary File S1 for full dataset). We found a total of 414 significantly upregulated genes (*P* < 0.05, log fold change > 0.5) and 222 significantly downregulated genes (*P* < 0.05, log fold change <−0.5). Using the CancerSEA resource (http://biocc.hrbmu.edu.cn/CancerSEA/home.jsp), differentially expressed genes were associated with angiogenesis, EMT, hypoxia, metastasis, invasion and stemness (see Supplementary file S2). Altered genes were also associated with a number of Gene Ontology (GO) terms with relevance to metastatic cancers including apoptosis, migration/cytoskeletal remodelling and response to stimuli (Fig. 3a and Supplementary File S2). KEGG pathway analysis suggested alterations in pathways associated with calcium signalling, glutamate signalling and circadian entrainment (Fig. 3b and Supplementary file S2). Many of the most significantly altered single genes have previously been identified in association with increased metastasis including decreases in genes such as MAGEA1, CPLX2 and KCND3 and increases in genes such as ROR2, HMGCS2, CAPG and SNCG (Fig. 3c).

### Tumour growth in vivo following intraductal injection of ILC cell lines

**Fig. 4:**
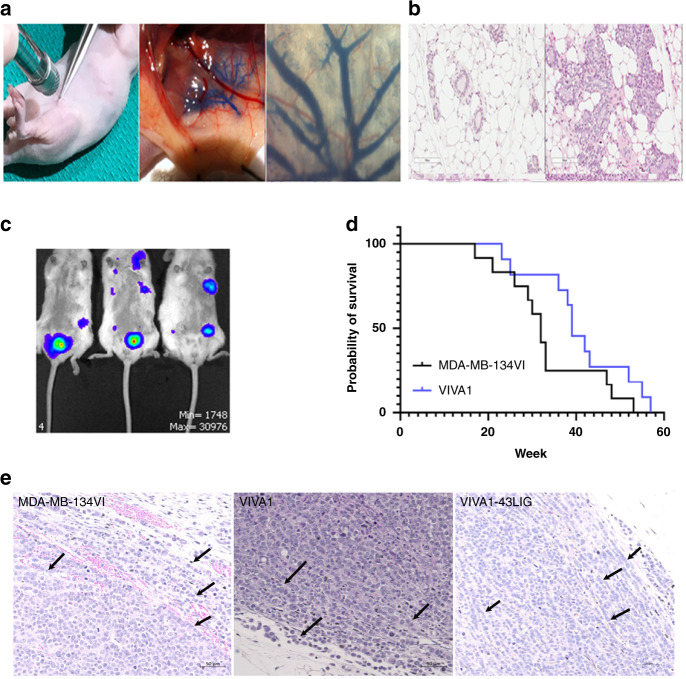
VIVA1 subclone and parental MDA-MB-134VI grow in immunocompromised animals following intraductal injection of tumour cells. **a** ILC Tumour cells were injected intraductally in immunocompromised mice via injection through the nipple, which resulted in the dissemination of injected material throughout the mammary ductal tree as evidenced by the distribution of Evan’s blue dye. **b** Intraductal injection of MDA-MB-134VI cells in CD-1 nude mice resulted in growth of tumours within the mammary ductal lumen by 24 weeks post injection as evident in H&E-stained tissue sections (right panel) compared to the appearance of normal ductal structures observed in uninjected mammary glands (left panel). **c** Growth of luciferase-expressing VIVA1 cells is evident in injected mammary glands by ~24–26 weeks post injection as detected by IVIS. **d** MDA-MB-134VI (12 animals), and VIVA1 (11 animals) were injected intraductally into the inguinal and abdominal mammary glands of NSG mice, and animals were euthanized upon reaching a clinical endpoint, with survival time recorded. Overall survival of mice was plotted against time. **e** Tumours arising following intraductal injection of MDA-MB-134VI parental cells, VIVA1 cells or the VIVA1-LIG43 primary tumour-derived cells were stained with H&E to visualise tumour cell histology and tumour growth patterns. Single-file growth patterns were observed along invasive edges of tumours for all three cell lines.

As there was evidence that MDA-MB-134VI could grow in mammary glands following intraductal injection of tumour cells [34], we established this procedure in our laboratory using the methods described above. It should be noted that in the article by Sflomos et al. [34], the authors reported that MDA-MB-134VI growth in mammary ducts was evident at ~20 weeks post injection, however they did not report assessment of tumour growth kinetics beyond this time frame in this original manuscript. To facilitate our analysis, we initially transduced MDA-MB-134VI and VIVA1 cells with vectors expressing luciferase in order to monitor tumour growth in vivo by IVIS. Upon confirmation of transduction and expression of luciferase in vitro, MDA-MB-134VI cells were injected intraductally (Fig. 4a). For initial studies, we injected 1 × 10^5^ MDA-MB-134VI cells intraductally into abdominal and inguinal mammary glands of CD-1 Nude mice, and after 24 weeks collected the mammary glands for histochemical analysis. As can be seen in Fig. 4b, MDA-MB-134VI cells readily grew within injected mammary ducts. Upon demonstrating, we could successfully establish tumours in mammary glands following intraductal administration of MDA-MB-134VI tumour cells, we decided to repeat experiments using NSG mice as it has been shown that the absence of NK cells in this animal strain facilitates more rapid tumour growth and metastasis of breast cancers [46]. Thus, we repeated intraductal injections of abdominal and inguinal mammary glands with MDA-MB-134VI (*n* = 12) or VIVA1 cells (*n* = 11) using the NSG mouse model. VIVA1 tumour growth in NSG mice was evident by ~26 weeks as detected by IVIS (Fig. 4c). Tumours were allowed to continue to grow to predefined clinical endpoints at which time animals were euthanized, autopsied, and time post injection of tumour cells noted for survival analysis. As seen in Fig. 4d, VIVA1 and MDA-MB-134VI had similar growth kinetics with median overall survival of 39 weeks and 32 weeks for VIVA1 and MDA-MB-134VI respectively, which was determined to not be statistically different using log-rank (Mantel–Cox) testing.

**Fig. 5:**
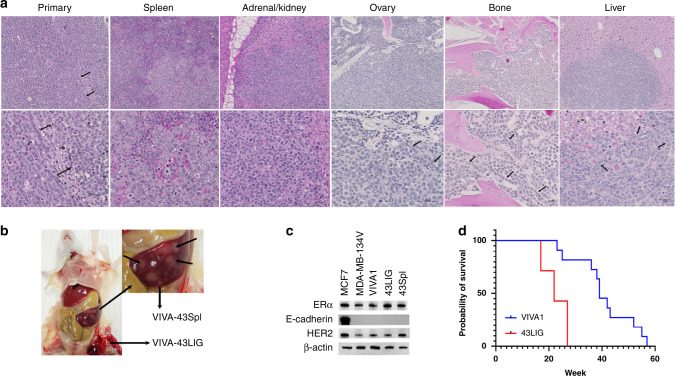
Intraductally injected VIVA1 cells generate tumours with features of ILC and spontaneously metastasize from the orthotopic site. At clinical endpoint, animals injected with ILC tumour cell lines were autopsied and portions of tumours retrieved for H&E histological assessment, while other portions were used to generate single-cell suspensions which were then cultured and expanded in vitro. **a** H&E staining of VIVA1 induced primary tumour and metastasis to spleen, adrenal gland, ovary, bone and liver were also observed with single-file growth patterns often observed at invasive edges or in the presence of additional stroma (indicated by arrows in each labelled panel). **b** Cells isolated from both primary tumour (43LIG) and the splenic metastasis (43Spl) of the same animal were expanded and protein lysates generated. **c** Western blot analysis of ILC markers showed the primary and metastases derived cell lines retained expression of ILC markers. MCF7 ductal carcinoma cells were included as a positive control for E-cadherin expression. β-actin was included as a loading control. **d** Cells isolated from a VIVA1 induced primary tumour (43LIG) were reinjected intraductally into additional animals (*n* = 6). Overall survival was plotted against the original survival of animals intraductally injected with VIVA1 cells previously shown in Fig. 4 for comparison.

Upon euthanasia and autopsy, we found that tumours grew in 100% of animals injected with VIVA1 or MDA-MB-134VI cells, although they may not necessarily have grown in all glands injected (normally 4–6 glands per animal), likely due to the difficult technical nature of intraductal injections. On average we observed successful tumour establishment and growth in ~64% of glands injected with MDA-MB-134VI cells and ~78% of glands injected with VIVA1 cells. At the endpoint, portions of the primary tumour and putative metastatic sites were fixed and embedded for H&E analysis while additional portions were used to isolate tumour cells as described in the Methods. The histological appearance of VIVA1 tumours showed similar growth patterns to MDA-MB-134VI tumours in vivo (Fig. 4e), with both tumour ‘islands’ possibly due to initial growth being confined to within the mammary ducts, and single-file cell growth patterns when tumours invaded surrounding stroma (Fig. 4e, arrows). We did not observe any obvious macrometastatic tumours in animals that had been injected with MDA-MB-134VI at the time of dissection. However, we observed spontaneous macrometastasis to various organs in 7/10 mice (one carcass was unable to be dissected) injected with VIVA1 cells with 3/7 mice having metastases in multiple sites concurrently. Sites of metastasis included the liver (2/7), bone (3/7), ovary (2/7), adrenal gland (1/7) and the spleen (1/7) (Fig. 5a). VIVA1 cell metastatic growth appeared to grow more in the island cell pattern, however, single-cell like growth patterns could also be seen in invading cell fronts (Fig. 5a, arrows). To confirm the identity of arising tumours, we assessed the expression of luciferase and ILC markers on cells isolated from both primary (VIVA-43LIG) and splenic metastases (VIVA-43Spl) from the same animal (Fig. 5b). In addition to expressing detectable levels of luciferase (not shown), cells isolated from tumour-bearing animals retained expression of ERα and Her2 at levels similar to both VIVA1 and parental MDA-MB-134VI cells and remained negative for E-cadherin (Fig. 5c). We also tested the ability of cells isolated from a VIVA1 induced primary tumour (VIVA-43LIG) for their ability to grow in vivo following re-injection intraductally. As seen in Fig. 5d, VIVA-43LIG cells grew faster in vivo than the original parental VIVA1 cells, with a median overall survival of 22 weeks (*n* = 6) suggesting a selection for cells with enhanced in vivo growth abilities by the original passaging of VIVA1 cells in the mouse mammary gland. VIVA-43LIG cells also appeared to form tumours in the mammary gland with similar histological patterns to those seen following injection of parental VIVA1 cells (Fig. 4e). At the endpoint, we found ~78% of injected mammary glands had extensive tumour growth, and macrometastases were observed in 4/6 animals injected with VIVA-43LIG cells (67%). Sites of metastasis were similar to that seen with VIVA1 parental cell injections with some animals showing multiple sites of metastatic tumour growth. Metastases were found in the spleen (1/4), kidney/adrenal gland (2/4), liver (1/4) and bone (2/4). These findings suggest that VIVA-43LIG cells have similar metastatic capabilities to VIVA1 cells but have more rapid primary tumour growth following intraductal injection in vivo.

**Fig. 6:**
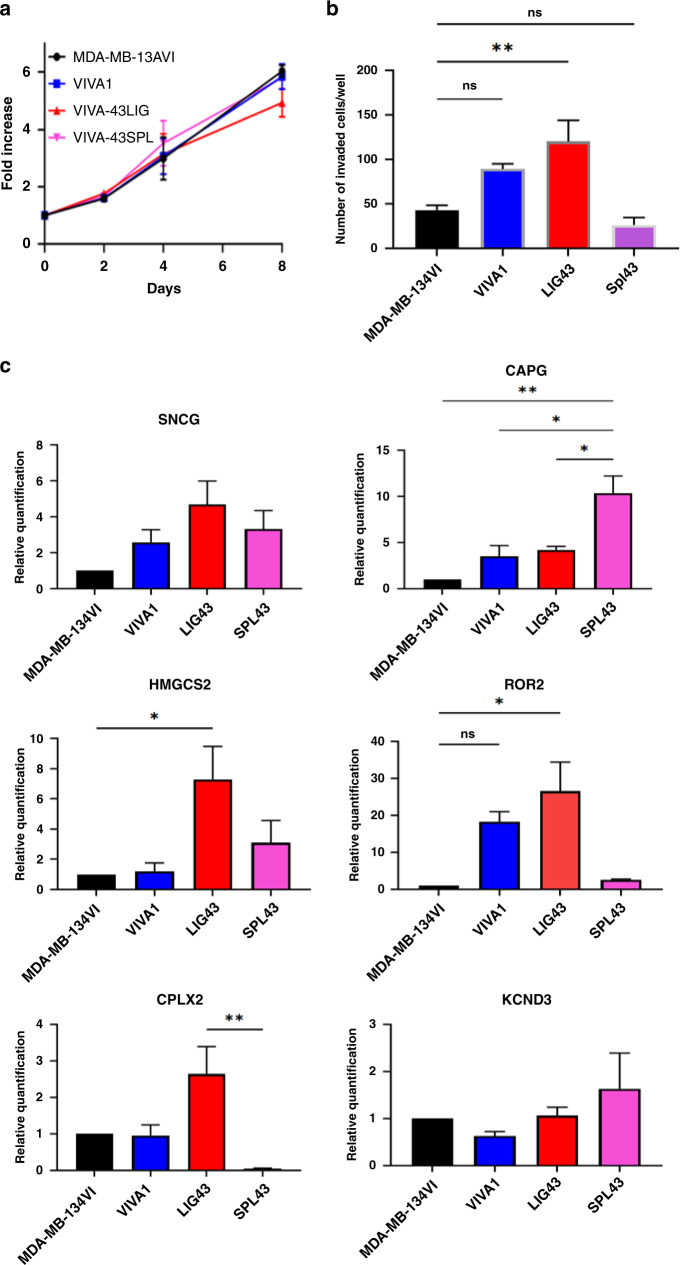
Cells isolated from VIVA1-derived primary or metastatic tumours retain increased invasive properties compared to parental MDA-MB-134VI cells. Cells were isolated from primary tumour or splenic metastatic sites from animals intraductally injected with VIVA1 cells and expanded in vitro. **a** Cell growth over time was measured in 2D in vitro culture following viable cell counting with AlamarBlue as described in 'Methods'. All cell lines tested showed similar growth kinetics with the graph representing the mean and standard error of eight technical replicates in each of three biological replicates. **b** Cell invasion was tested using Matrigel-coated invasion chambers and incubation for 7 days, at which time membranes were isolated, stained and invaded cells counted as described in 'Methods'. Graph is the mean and standard error of duplicate wells in each of three independent biological replicates. VIVA1 (*P* = 0.051) and primary tumour-derived VIVA1-LIG43 cells retained increased invasive abilities compared to MDA-MB-134VI parental cells. **P value <0.001. **c** RNA isolated from the various cell lines was subjected to qRT-PCR for highly differentially expressed genes identified in previous RNA-seq experiments. Graphs represent mean and standard error for relative levels of expression of various targets as indicated following normalisation to levels of β-actin as a control from three technical replicates for each of three independent biological replicates. *P value < 0.05; **P value <0.001.

To better characterise VIVA1-derived cell lines isolated from in vivo tumours, we compared the tumour cell growth and invasion in vitro. We found no significant proliferative difference between the primary tumour-derived VIVA-43LIG or metastasis-derived VIVA-43Spl cells compared to VIVA1 or MDA-MB-134VI original parental cells in 2D culture in vitro (Fig. 6a). We confirmed that VIVA-43LIG cells had significantly increased cell invasion compared to the original parental MDA-MB-134VI cells (with VIVA1 also showing increased cell invasion that almost reached statistical significance in this case, *P* = 0.051), however, noted that VIVA-43Spl cells had similar invasion rates to the parental MDA-MB-134VI cells. We also examined whether any of the top differentially expressed genes identified by RNA-seq as illustrated in Fig. 3c could be validated and were associated with increased invasion or metastatic potential in these cells. We found trends for increased SNCG in the more invasive VIVA1, VIVA-43LIG and VIVA-43Spl cells compared to parental MDA-MB-134VI cells as predicted by the RNA-seq. We also observed corresponding trends for CAPG with increased levels in VIVA1 and VIVA-43LIG cells and statistically significant higher levels of CAPG in VIVA-Spl43 cells compared to parental MDA-MB-134VI cells. HMGCS2 was highest in VIVA-43LIG cells (*P*<0.05 compared to MDA-MB-134VI cells) and showed trends for higher expression in VIVA1 and VIVA-43Spl cells. We observed increased levels of ROR2 in VIVA1 and VIVA-43LIG cells compared to parental MDA-MB-134VI cells, however, ROR2 levels in VIVA-43Spl were substantially lower than that found in VIVA1 and VIVA-43LIG cells. For downregulated genes identified by RNA-seq, we were not able to validate MAGE1 or KCND3, in part due to their low abundance and difficulty to reliably detect by qRT-PCR. For CPLX2 we confirmed slight but statistically insignificant downregulation in VIVA1 compared to MDA-MB-134VI however VIVA-43LIG cells showed higher levels of CPLX2 compared to VIVA1 or MDA-MB-134VI cells. Although unexpected, it is interesting to note that CPLX2 was significantly downregulated in the VIVA-43Spl cells derived from a metastasis compared to the VIVA-43LIG cells isolated from a primary tumour in the same mouse, which does support an association of its downregulation with the promotion of metastatic growth. More recently we have isolated cell lines from additional sites of metastasis of VIVA1 cells including additional lines from the spleen, and new lines from the adrenal gland, ovary, liver and bones. Upon further characterisation and confirmation of tumour cell identity, these lines can be used to further validate and characterize the roles of candidate genes driving ILC metastasis in this model.

## Discussion

Due to the scarcity and limitations of existing ILC models that make studying ILC metastasis difficult, we attempted to derive invasive ILC cell lines with enhanced metastatic ability that could be used to generate new metastatic ILC xenograft models. To this end, we have generated a more invasive subclone of MDA-MB-134VI, the VIVA1 cell line, which maintained ILC marker expression and in vivo growth kinetics similar to the parental line but has increased ability to spontaneously metastasize from the orthotopic site in vivo.

In our initial studies assessing the invasive ability of ILC cell lines at our disposal, we found that MDA-MB-330 cells invaded quite readily at 48 h and 7 days post seeding of Matrigel-coated transwells, a finding that is in contrast with that of Tasdemir et al [24]. However, the lack of significant cell invasion with MDA-MB-134VI cells is similar to their findings. Methods of Tasdemir et al. differed from ours in two significant ways: the ratio of FBS in top versus bottom chambers differed and the culture medium for MDA-MB-330 was different between the two studies, where the media used by Tasemir et al., only contained 10% FBS and lacked the additional supplements in which we cultured MDA-MB-330 as described in the Methods section. We tested MDA-MB-330 invasion using the same FBS ratios as utilised by Tasemir et al. but retaining them in our routinely used culture media with additional supplements and found that they still readily invaded at 48 h (data not shown), thus speculate that MDA-MB-330 growth in the additional supplements recommended by ATCC confers increased invasive abilities. While we have not formally demonstrated the reason for the increased invasion of MDA-MB-134VI cells at 7 days post seeding compared to 48 h post seeding we speculate it is likely due to both additional invading cells over time and possible proliferation of cells that have already invaded early post seeding. Images of stained invasion membranes mostly had single cells visible but in some cases cell doublets could be observed (not shown) thus it is feasible cells could proliferate given the reported doubling time of MDA-MB-134VI cells ranges from 4 to 6 days [24, 47]. However, as VIVA1 cells had similar in vitro proliferation rates as the parental MDA-MB-134VI cells, we believe the increased cell invasion observed in Fig. 2 is predominantly due to increased invasive abilities of the VIVA1 subclone.

In vivo growth of MDA-MB-134VI in our hands was similar to that originally reported by Sflomos et al. [34], who reported an 80% engraftment rate of intraductally injected MDA-MB-134VI cells, and an 83% engraftment rate in a more recent study where four mammary ducts per mouse were injected [48]. In our case, 100% of injected mice exhibited successful engraftment and tumour growth, however when we routinely injected 4–6 mammary ductal trees per mouse, we observed ~75–80% of injected glands developing growing tumours by endpoint. We saw similar engraftment rates for the VIVA1 cell line, which was derived as a more invasive subclone of MDA-MB-134VI. Although the median survival of VIVA1-injected animals was slightly longer than those injected with MDA-MB-134VI, this was not statistically significant. Given the similar proliferation rates between VIVA1 and MDA-MB-134VI in vitro, it is not surprising they showed similar tumour growth rates and overall survival in vivo, as for the most part the clinical endpoints reached were due to reaching limits of allowable primary tumour burden. It should be noted that the current model assessed tumour growth in the absence of exogenous oestrogen. It has been shown that implantation of oestrogen releasing pellets can increase ILC tumour cell growth in vivo and decrease tumour latency in a PDX model [43], thus we speculate that the addition of exogenous oestrogen sources could reduce tumour latency in the VIVA1 model, however, this has not yet been tested. Since ILC is fairly slow growing in patients, the long latency of this model is perhaps reflective of the nature of this cancer type in patients and is of benefit. Despite the long latency, it is exciting to see that VIVA1 cells appear to have greater invasive abilities not only in vitro, but in vivo as well given our ability to detect macrometastases in 70% of animals at endpoint. It should be noted that the spleen, liver, bone, adrenal gland and ovary are relevant metastatic sites reported to occur in ILC patients [16, 49–52] and were also metastatic sites reported in similar models using MDA-MB-134VI parental cells [48]. Although we did not see macrometastatic burden in mice injected with MDA-MB-134VI cells in our study, it should be noted that we injected approximately fivefold fewer cells per mammary gland than was used in the recently reported study using similar approaches [48]. We also did not observe macrometastatic lesions in the lungs following VIVA1 cell injection, while evidence of metastasis to lungs was shown for the parental cell line in the previously published MDA-MB-134VI model [48]. The lack of metastasis to the lung in the VIVA1 model could be due in part to the use of reduced primary tumour cell inoculation as mentioned, or it remains possible that in vitro selection of highly invasive clones has resulted in the preclusion of metastasis to the lungs. Notably, however, VIVA1 cells do metastasize to all the other sites noted by Sflomos et al and thus remains a useful tool to study the metastatic process in ILC. Along with parental MDA-MB-134VI cells and the ER+ HCI-013 ILC PDX model which has been reported to metastasize to lungs (but not to any of the other sites our model appears to) [53] the VIVA1 model will contribute to our increased understanding of factors driving ILC metastasis and provide an additional validation tool to existing metastatic ILC in vivo models.

Although both MDA-MB-134VI and VIVA1 tumours in vivo generally appeared to grow in cell islands, they also showed features of the hallmark ILC growth pattern of aligned single-file cells (Fig. 4e) particularly at the invasive edges. Similar growth patterns were recently reported for the parental MDA-MB-134VI cells and it was suggested this could be due to the fact that these cells harbour p53 mutations which are usually associated with the pleomorphic subtype of ILC which show similar growth patterns in vivo [48]. We speculate that the growth pattern observed in primary tumours may also be due to the nature of the administration of cells within the ductal lumen space where additional stromal components and ECM would be lacking. This is supported by the evidence of the ability of the cells to grow in the ‘single file’ cell pattern often observed in classical ILC patient tumours upon invasion into areas of the mammary gland which contain more stroma and suggests this pattern of growth could be induced by the presence of stroma as opposed to the intrinsic tumour cell phenotype directly. In fact, there are variations in the growth pattern of classical ILC, with island cell growth patterns previously reported in patients [54, 55]. Notably, VIVA1 tumours histologically appeared similar to those generated following intraductal administration of MDA-MB-134VI [48] and fatpad implantation of the metastatic ER+ ILC PDX model HCI-0013, which also showed growth in cell islands [43]. As such the growth pattern of VIVA1 tumour cells in vivo is reflective of ILC growth pattern observed in patients and other commonly used ILC xenograft models. Although our model is limited in the context of being a xenograft model, this limitation could be overcome in future studies with the current availability of CD34+ humanised mice which would allow assessment of the role of the immune response in the metastatic spread from the orthotopic site. Although the VIVA1 model is derived from a single pooled cell subpopulation, the ease of its use for in vitro analyses which can be validated in the in vivo xenograft will help facilitate research in metastatic ILC which could then be validated in other available model systems.

Although characterisation of the VIVA1 model is still in its infancy, initial RNA-seq data has led to tantalising clues supporting putative mechanisms driving their increased invasive and metastatic ability. We uncovered significant differences in gene expression levels of factors associated with decreased metastasis and better prognosis, such as decreases in MAGEA1, which was shown to decrease breast cancer cell migration and invasion via its ability to promote FBXW7-mediated Notch receptor degradation [56], and whose increased expression is associated with better prognosis in breast cancer [57]. We also found significant decreases of CPLX2, which is decreased during the progression of oesophageal tumours [58] and decreased in association with lymphatic invasion in neuroendocrine lung tumours [59], and KCND3 whose levels decrease in association with increased breast cancer stage [60] in VIVA1 cells compared to the parental MDA-MB-134VI cell line. We attempted to validate these findings in the VIVA-43LIG and VIVA-43Spl cell lines using qRT-PCR however found that transcript levels were too low to be reliably detected using this method. We did however confirm that levels of CPLX2 were significantly lower in the metastasis-derived VIVA1-43Spl cell line compared to the primary tumour-derived VIVA1-43LIG cell line derived from the same animal (Fig. 6c) supporting a role for its downregulation in association with metastasis. We also saw significant upregulation of many genes with notable associations to metastasis such as ROR2 whose overexpression was shown to promote breast cancer cell invasion [61, 62] and increased expression was associated with worse overall survival in breast cancer patients [62]. Other genes significantly increased in VIVA1 cells with known links to metastasis included HMGCS2, which increased tumour cell invasion and metastasis in oral and colorectal cancer [63] as well as breast cancer cell lines [64], and SNCG which has also been shown to increase breast cancer cell invasion and metastasis when overexpressed [65, 66]. Lastly, CAPG was significantly elevated in VIVA1 cells and has recently been shown to promote breast cancer metastasis via its ability to influence epigenetic modifications in cancer cells [67] and was prognostic for development of metastasis in adjuvant-treated breast cancer patients [68]. With respect to these upregulated genes, we could validate significant increases in expression levels for HMGCS2 and CAPG in VIVA-43LIG and VIVA-43Spl cells for the former, with trends for upregulated expression of SNCG in VIVA-43LIG and VIVA-43Spl cells (Fig. 6c) supporting their putative role in driving metastasis of ILC. We also found significantly increased expression of ROR2 in VIVA-43LIG cells, however, ROR2 levels were reduced in VIVA-43Spl cells. This could be a reflection of its proposed role in increasing invasive properties of cells, which is subsequently dispensable for the growth of already disseminated tumour cells in progressing metastases. This reduced level of expression of ROR2 and its association with increased invasion is also supported by our findings that VIVA-43Spl cells have reduced cell invasion in vitro compared to VIVA-43LIG cells (Fig. 6b).

KEGG pathway analysis of RNA-seq datasets suggested significant associations with calcium signalling, glutamate signalling and circadian entrainment. It appears the identified associated pathways may be driven by differential expression of the same subset of genes which are found in all three pathways, including CACNA1C, ADCY1, GRIN1 and PLCB2. Although their specific role in ILC metastasis in our model remains to be examined, it is interesting to note that some of these targets such as ADCY1, which was decreased in VIVA1 compared to parental cells, have been recently shown to be decreased in association with increased metastasis in other tumour types [69, 70], and PLCB2 which was increased in VIVA1 cells has been associated with worse overall survival in lung cancer [71]. It will be important to examine the roles of these factors in driving ILC metastasis in the VIVA1 model and additional in vivo metastatic ILC models in future studies.

In conclusion, we isolated a more invasive subclone from the representative ILC cell line MDA-MB-134VI which we named VIVA1. VIVA1 cells showed similar proliferative rates, but increased invasion compared to parental cells in vitro and readily grew orthotopically following intraductal administration of tumour cells with 70% of animals developing spontaneous metastasis to common sites of metastases found in ILC patients. The development of the VIVA1 metastatic ILC xenograft model will facilitate the identification of targets driving ILC metastasis and will be useful to test whether therapeutic blockade of these targets could be efficacious in ILC patients in the future.

### Reporting summary

Further information on research design is available in the Nature Research Reporting Summary linked to this article.

## Supplementary information

Legends for Supplemental Files

Supplemental File S1

Supplemental File S2

Author Checklist

Author Checklist - Reporting Summary

## References

[1] Iorfida M, Maiorano E, Orvieto E, Maisonneuve P, Bottiglieri L, Rotmensz N (2012). Invasive lobular breast cancer: subtypes and outcome. Breast Cancer Res Treat.

[2] Nielsen TO, Parker JS, Leung S, Voduc D, Ebbert M, Vickery T (2010). A comparison of PAM50 intrinsic subtyping with immunohistochemistry and clinical prognostic factors in tamoxifen-treated estrogen receptor-positive breast cancer. Clin Cancer Res.

[3] Hugh J, Hanson J, Cheang MC, Nielsen TO, Perou CM, Dumontet C (2009). Breast cancer subtypes and response to docetaxel in node-positive breast cancer: use of an immunohistochemical definition in the BCIRG 001 trial. J Clin Oncol.

[4] Cheang MC, Chia SK, Voduc D, Gao D, Leung S, Snider J (2009). Ki67 index, HER2 status, and prognosis of patients with luminal B breast cancer. J Natl Cancer Inst.

[5] Weigelt B, Geyer FC, Natrajan R, Lopez-Garcia MA, Ahmad AS, Savage K (2010). The molecular underpinning of lobular histological growth pattern: a genome-wide transcriptomic analysis of invasive lobular carcinomas and grade- and molecular subtype-matched invasive ductal carcinomas of no special type. J Pathol.

[6] Yoder BJ, Wilkinson EJ, Massoll NA (2007). Molecular and morphologic distinctions between infiltrating ductal and lobular carcinoma of the breast. Breast J.

[7] Yeatman TJ, Cantor AB, Smith TJ, Smith SK, Reintgen DS, Miller MS (1995). Tumor biology of infiltrating lobular carcinoma. Implications for management. Ann Surg.

[8] Cristofanilli M, Gonzalez-Angulo A, Sneige N, Kau SW, Broglio K, Theriault RL (2005). Invasive lobular carcinoma classic type: response to primary chemotherapy and survival outcomes. J Clin Oncol.

[9] Wasif N, Maggard MA, Ko CY, Giuliano AE (2010). Invasive lobular vs. ductal breast cancer: a stage-matched comparison of outcomes. Ann Surg Oncol.

[10] Cortazar P, Zhang L, Untch M, Mehta K, Costantino JP, Wolmark N, et al. Pathological complete response and long-term clinical benefit in breast cancer: the CTNeoBC pooled analysis. Lancet. 2014. 10.1016/s0140-6736(13)62422-8. 10.1016/S0140-6736(13)62422-8 24529560

[11] Loibl S, Volz C, Mau C, Blohmer JU, Costa SD, Eidtmann H (2014). Response and prognosis after neoadjuvant chemotherapy in 1,051 patients with infiltrating lobular breast carcinoma. Breast Cancer Res Treat.

[12] Voogd AC, Nielsen M, Peterse JL, Blichert-Toft M, Bartelink H, Overgaard M (2001). Differences in risk factors for local and distant recurrence after breast-conserving therapy or mastectomy for stage I and II breast cancer: pooled results of two large European randomized trials. J Clin Oncol.

[13] Hadji P, Coleman R, Gnant M, Green J (2012). The impact of menopause on bone, zoledronic acid, and implications for breast cancer growth and metastasis. Ann Oncol.

[14] Suva LJ, Griffin RJ, Makhoul I (2009). Mechanisms of bone metastases of breast cancer. Endocr-Relat Cancer.

[15] Korhonen T, Kuukasjarvi T, Huhtala H, Alarmo EL, Holli K, Kallioniemi A (2013). The impact of lobular and ductal breast cancer histology on the metastatic behavior and long term survival of breast cancer patients. Breast.

[16] Ferlicot S, Vincent-Salomon A, Medioni J, Genin P, Rosty C, Sigal-Zafrani B (2004). Wide metastatic spreading in infiltrating lobular carcinoma of the breast. Eur J Cancer.

[17] Dabbs DJ, Schnitt SJ, Geyer FC, Weigelt B, Baehner FL, Decker T (2013). Lobular neoplasia of the breast revisited with emphasis on the role of E-cadherin immunohistochemistry. Am J Surg Pathol.

[18] Moll R, Mitze M, Frixen UH, Birchmeier W (1993). Differential loss of E-cadherin expression in infiltrating ductal and lobular breast carcinomas. Am J Pathol.

[19] Derksen PW, Liu X, Saridin F, van der Gulden H, Zevenhoven J, Evers B (2006). Somatic inactivation of E-cadherin and p53 in mice leads to metastatic lobular mammary carcinoma through induction of anoikis resistance and angiogenesis. Cancer Cell.

[20] Ciriello G, Gatza ML, Beck AH, Wilkerson MD, Rhie SK, Pastore A (2015). Comprehensive molecular portraits of invasive lobular breast cancer. Cell.

[21] Desmedt C, Zoppoli G, Sotiriou C, Salgado R (2017). Transcriptomic and genomic features of invasive lobular breast cancer. Semin Cancer Biol.

[22] Chen Z, Yang J, Li S, Lv M, Shen Y, Wang B (2017). Invasive lobular carcinoma of the breast: a special histological type compared with invasive ductal carcinoma. PLoS ONE.

[23] Cortazar P, Zhang L, Untch M, Mehta K, Costantino JP, Wolmark N (2014). Pathological complete response and long-term clinical benefit in breast cancer: the CTNeoBC pooled analysis. Lancet.

[24] Tasdemir N, Bossart EA, Li Z, Zhu L, Sikora MJ, Levine KM (2018). Comprehensive phenotypic characterization of human invasive lobular carcinoma cell lines in 2D and 3D cultures. Cancer Res.

[25] Christgen M, Noskowicz M, Heil C, Schipper E, Christgen H, Geffers R (2012). IPH-926 lobular breast cancer cells harbor a p53 mutant with temperature-sensitive functional activity and allow for profiling of p53-responsive genes. Lab Investig.

[26] An Y, Adams JR, Hollern DP, Zhao A, Chang SG, Gams MS (2018). Cdh1 and Pik3ca mutations cooperate to induce immune-related invasive lobular carcinoma of the breast. Cell Rep..

[27] Boelens MC, Nethe M, Klarenbeek S, de Ruiter JR, Schut E, Bonzanni N (2016). PTEN loss in E-cadherin-deficient mouse mammary epithelial cells rescues apoptosis and results in development of classical invasive lobular carcinoma. Cell Rep..

[28] Derksen PW, Braumuller TM, van der Burg E, Hornsveld M, Mesman E, Wesseling J (2011). Mammary-specific inactivation of E-cadherin and p53 impairs functional gland development and leads to pleomorphic invasive lobular carcinoma in mice. Dis Models Mechanisms.

[29] Guillen KP, Fujita M, Butterfield AJ, Scherer SD, Bailey MH, Chu Z (2022). A human breast cancer-derived xenograft and organoid platform for drug discovery and precision oncology. Nat Cancer.

[30] Cailleau R, Young R, Olive M, Reeves WJ (1974). Breast tumor cell lines from pleural effusions. J Natl Cancer Inst.

[31] Neve RM, Chin K, Fridlyand J, Yeh J, Baehner FL, Fevr T (2006). A collection of breast cancer cell lines for the study of functionally distinct cancer subtypes. Cancer Cell.

[32] Reis-Filho JS, Simpson PT, Turner NC, Lambros MB, Jones C, Mackay A (2006). FGFR1 emerges as a potential therapeutic target for lobular breast carcinomas. Clin Cancer Res.

[33] Christgen M, Derksen P (2015). Lobular breast cancer: molecular basis, mouse and cellular models. Breast Cancer Res: BCR.

[34] Sflomos G, Dormoy V, Metsalu T, Jeitziner R, Battista L, Scabia V (2016). A preclinical model for ERalpha-positive breast cancer points to the epithelial microenvironment as determinant of luminal phenotype and hormone response. Cancer Cell.

[35] Christgen M, Bruchhardt H, Hadamitzky C, Rudolph C, Steinemann D, Gadzicki D (2009). Comprehensive genetic and functional characterization of IPH-926: a novel CDH1-null tumour cell line from human lobular breast cancer. J Pathol.

[36] Bray NL, Pimentel H, Melsted P, Pachter L (2016). Near-optimal probabilistic RNA-seq quantification. Nat Biotechnol.

[37] Pimentel H, Bray NL, Puente S, Melsted P, Pachter L (2017). Differential analysis of RNA-seq incorporating quantification uncertainty. Nat Methods.

[38] Lim E, Modi K, Christensen A, Meganck J, Oldfield S, Zhang N. Monitoring tumor metastases and osteolytic lesions with bioluminescence and micro CT imaging. J Vis Exp: JoVE. 2011; 10.3791/2775. 10.3791/2775 PMC3169249 21525842

[39] Canon JR, Roudier M, Bryant R, Morony S, Stolina M, Kostenuik PJ (2008). Inhibition of RANKL blocks skeletal tumor progression and improves survival in a mouse model of breast cancer bone metastasis. Clin Exp Metastasis.

[40] Fischer AH, Jacobson KA, Rose J, Zeller R. Hematoxylin and eosin staining of tissue and cell sections. CSH Protoc. 2008;2008:pdb prot4986. 10.1101/pdb.prot4986 21356829

[41] Sottnik JL, Bordeaux EK, Mehrotra S, Ferrara SE, Goodspeed AE, Costello JC, et al. Mediator of DNA damage checkpoint 1 (MDC1) is a novel estrogen receptor co-regulator in invasive lobular carcinoma of the breast. Mol Cancer Res: MCR. 2021; 10.1158/1541-7786.MCR-21-0025. 10.1158/1541-7786.MCR-21-0025 PMC8349796 33947745

[42] Du T, Sikora MJ, Levine KM, Tasdemir N, Riggins RB, Wendell SG (2018). Key regulators of lipid metabolism drive endocrine resistance in invasive lobular breast cancer. Breast Cancer Res: BCR.

[43] Sikora MJ, Cooper KL, Bahreini A, Luthra S, Wang G, Chandran UR (2014). Invasive lobular carcinoma cell lines are characterized by unique estrogen-mediated gene expression patterns and altered tamoxifen response. Cancer Res.

[44] Levine KM, Priedigkeit N, Basudan A, Tasdemir N, Sikora MJ, Sokol ES (2019). FGFR4 overexpression and hotspot mutations in metastatic ER+ breast cancer are enriched in the lobular subtype. NPJ Breast Cancer.

[45] Tasdemir N, Bossart EA, Li Z, Zhu L, Sikora MJ, Levine KM, et al. Comprehensive phenotypic characterization of human invasive lobular carcinoma cell lines in 2D and 3D cultures. Cancer Res. 2018; 10.1158/0008-5472.CAN-18-1416. 10.1158/0008-5472.CAN-18-1416 PMC6507416 30228172

[46] Puchalapalli M, Zeng X, Mu L, Anderson A, Hix Glickman L, Zhang M (2016). NSG mice provide a better spontaneous model of breast cancer metastasis than athymic (Nude) mice. PLoS ONE.

[47] Wang J, Xie X, Sun Y (1998). Lack of relationship between CDK activity and G1 cyclin expression in breast cancer cells. Oncogene..

[48] Sflomos G, Battista L, Aouad P, De Martino F, Scabia V, Stravodimou A (2021). Intraductal xenografts show lobular carcinoma cells rely on their own extracellular matrix and LOXL1. EMBO Mol Med.

[49] Groisman GM (2016). Lobular carcinoma of the breast metastatic to the spleen and accessory spleen: report of a case. Case Rep. Pathol.

[50] Hasadia R, Kazarin O, Sofer O, Shulman K, Troitsa A, Alfici R, et al. Splenectomy for breast carcinoma diffusely metastatic to the spleen presenting as severe transfusion-dependent anaemia and thrombocytopaenia. BMJ Case Rep. 2018;11:e223453. 10.1136/bcr-2017-223453 PMC6303665 30567891

[51] Lamovec J, Bracko M (1991). Metastatic pattern of infiltrating lobular carcinoma of the breast: an autopsy study. J Surg Oncol.

[52] Mathew A, Rajagopal PS, Villgran V, Sandhu GS, Jankowitz RC, Jacob M (2017). Distinct pattern of metastases in patients with invasive lobular carcinoma of the breast. Geburtshilfe und Frauenheilkd.

[53] Williams MM, Spoelstra NS, Arnesen S, O’Neill KI, Christenson JL, Reese J (2021). Steroid hormone receptor and infiltrating immune cell status reveals therapeutic vulnerabilities of ESR1-mutant breast cancer. Cancer Res.

[54] McCart Reed AE, Kutasovic JR, Lakhani SR, Simpson PT (2015). Invasive lobular carcinoma of the breast: morphology, biomarkers and ‘omics. Breast Cancer Res: BCR.

[55] Dixon JM, Anderson TJ, Page DL, Lee D, Duffy SW (1982). Infiltrating lobular carcinoma of the breast. Histopathology.

[56] Zhao J, Wang Y, Mu C, Xu Y, Sang J (2017). MAGEA1 interacts with FBXW7 and regulates ubiquitin ligase-mediated turnover of NICD1 in breast and ovarian cancer cells. Oncogene.

[57] Jia B, Zhao X, Wang Y, Wang J, Yang Y (2019). Prognostic roles of MAGE family members in breast cancer based on KM-Plotter Data. Oncol Lett.

[58] Wang J, Xie X, Sun Y (2020). Time series expression pattern of key genes reveals the molecular process of esophageal cancer. Biosci Rep..

[59] Komatsu H, Kakehashi A, Nishiyama N, Izumi N, Mizuguchi S, Yamano S (2013). Complexin-2 (CPLX2) as a potential prognostic biomarker in human lung high grade neuroendocrine tumors. Cancer Biomark: Sect A Dis Mark.

[60] Ko JH, Ko EA, Gu W, Lim I, Bang H, Zhou T (2013). Expression profiling of ion channel genes predicts clinical outcome in breast cancer. Mol Cancer.

[61] Bayerlova M, Menck K, Klemm F, Wolff A, Pukrop T, Binder C (2017). Ror2 signaling and its relevance in breast cancer progression. Front Oncol.

[62] Menck K, Heinrichs M, Wlochowitz D, Sitte M, Noeding H, Janshoff A (2021). WNT11/ROR2 signaling is associated with tumor invasion and poor survival in breast cancer. J Exp Clin Cancer Res..

[63] Chen SW, Chou CT, Chang CC, Li YJ, Chen ST, Lin IC (2017). HMGCS2 enhances invasion and metastasis via direct interaction with PPARalpha to activate Src signaling in colorectal cancer and oral cancer. Oncotarget.

[64] Martinez-Outschoorn UE, Lin Z, Whitaker-Menezes D, Howell A, Sotgia F, Lisanti MP (2012). Ketone body utilization drives tumor growth and metastasis. Cell Cycle.

[65] Jia T, Liu YE, Liu J, Shi YE (1999). Stimulation of breast cancer invasion and metastasis by synuclein γ. Cancer Res.

[66] Zhuang Q, Liu C, Qu L, Shou C (2015). Synuclein-γ promotes migration of MCF7 breast cancer cells by activating extracellular-signal regulated kinase pathway and breaking cell-cell junctions. Mol Med Rep..

[67] Huang S, Chi Y, Qin Y, Wang Z, Xiu B, Su Y (2018). CAPG enhances breast cancer metastasis by competing with PRMT5 to modulate STC-1 transcription. Theranostics.

[68] Westbrook JA, Cairns DA, Peng J, Speirs V, Hanby AM, Holen I, et al. CAPG and GIPC1: breast cancer biomarkers for bone metastasis development and treatment. J Natl Cancer Inst. 2016;108:djv360. 10.1093/jnci/djv360 PMC4808632 26757732

[69] Hua Y, Ma X, Liu X, Yuan X, Qin H, Zhang X (2017). Identification of the potential biomarkers for the metastasis of rectal adenocarcinoma. APMIS: Acta pathologica, microbiologica, et immunologica Scandinavica.

[70] Zhang Y, Yang J, Wang X, Li X (2021). GNG7 and ADCY1 as diagnostic and prognostic biomarkers for pancreatic adenocarcinoma through bioinformatic-based analyses. Sci Rep..

[71] Zhang T, Song X, Liao X, Wang X, Zhu G, Yang C (2019). Distinct prognostic values of phospholipase C beta family members for non-small cell lung carcinoma. BioMed Res Int.

