# Supplementary material for: VIVA1: a more invasive subclone of MDA-MB-134VI invasive lobular carcinoma cells with increased metastatic potential in xenograft models
Source: Br J Cancer. 2022 Mar 22;127(1):56–68. doi: 10.1038/s41416-022-01778-7 (PMC9276762; doi:10.1038/s41416-022-01778-7)
Supplement: Supplementary file 1 — Legends for Supplemental Files [file 41416_2022_1778_MOESM1_ESM.docx]

Legends Accompanying Supplementary Files

Supplemental S1. Differential Gene Expression detected by RNAseq for VIVA1 compared to MDA-MB-134VI cells.

RNA was isolated from VIVA1 or MDA-MB-134VI cells and used to generate cDNA libraries which were then sequenced on the Illumina NextSeq 500 with paired end 42bp × 42bp reads. Gene assignment and transcript quantification from raw data was performed using Kallisto (v0.45.0) as described in Materials and Methods, and the R package Sleuth (v0.30.0) used to model gene expression changes between the cell lines with Wald’s testing performed to test for significant differential expression. Log2-fold difference in expression of each gene is indicated in column labeled ‘b’, with associated SEM indicated in column se_b, and p- and q-values indicated as labeled.

Supplemental S2. Pathway and Term analysis of statistically significant differentially expressed genes in VIVA1 compared to MDA-MB-134VI.

Gene lists containing those genes with log2-fold difference <-0.5 or >0.5 with associated p-values <0.05 were generated and submitted for analysis using: Sheet 1- CancerSEA analysis <http://biocc.hrbmu.edu.cn/CancerSEA/home.jsp>; Sheet 2 -KEGG pathway analysis <https://david.ncifcrf.gov/>; or sheet 3 - GO TERM BP analysis <https://david.ncifcrf.gov/>.
